# Chemopreventive Potential of Ethanolic Extracts of Luobuma Leaves (*Apocynum venetum* L.) in Androgen Insensitive Prostate Cancer

**DOI:** 10.3390/nu9090948

**Published:** 2017-08-28

**Authors:** Szu-Ping Huang, Tzu-Ming Ho, Chih-Wen Yang, Ya-Ju Chang, Jie-Fu Chen, Ning-Sing Shaw, Jia-Cherng Horng, Shih-Lan Hsu, Ming-Yuan Liao, Li-Chen Wu, Ja-an Annie Ho

**Affiliations:** 1 Department of Chemistry, National Tsing Hua University, Hsinchu 30013, Taiwan; 38903@yungshingroup.com (S.-P.H.); jchorng@mx.nthu.edu.tw (J.-C.H.); 2 BioAnalytical Chemistry and Nanobiomedicine Laboratory, Department of Biochemical Science and Technology, National Taiwan University, Taipei 10617, Taiwan; tzumingho@ntu.edu.tw (T.-M.H.); r02b22032@ntu.edu.tw (C.-W.Y.); d00b22001@ntu.edu.tw (Y.-J.C.); jeffwizardxd@gmail.com (J.-F.C.); 3 Applied and Translational Nutrition Laboratory, Department of Biochemical Science and Technology, National Taiwan University, Taipei 10617, Taiwan; nsshaw@ntu.edu.tw; 4 Department of Applied Chemistry, National Chi Nan University, Puli, Nantou 54561, Taiwan; 5 Department of Medical Education & Research, Taichung Veterans General Hospital, Taichung 40705, Taiwan; hsu2326@gmail.com; 6 Department of Chemistry, National Chung-Hsin University, Taichung 40227, Taiwan

**Keywords:** composition analysis, *Apocynum venetum* L., anti-cancer activity, synergistic therapy, lupeol, androgen-insensitive prostate cancer

## Abstract

Luobuma (*Apocynum venetum* L. (AVL)) is a popular beverage in Asia and has been reportedly to be associated with the bioactivities such as cardiotonic, diuretic, antioxidative, and antihypertensive. However, its biofunction as chemoprevention activity is seldom addressed. Herein, we aimed to characterize the anti-androgen-insensitive-prostate-cancer (anti-AIPC) bioactive compounds of Luobuma, and to investigate the associated molecular mechanisms. Activity-guided-fractionation (antioxidative activity and cell survivability) of Luobuma ethanolic extracts was performed to isolate and characterize the major bioactive compounds using Ultra Performance Liquid Chromatography (UPLC), Liquid Chromatography-Mass Spectrometry (LC-MS), and Nuclear Magnetic Resonance (NMR). Plant sterols (lupeol, stigamasterol and β-sitosterol) and polyphenolics (isorhamnetin, kaempferol, and quercetin) were identified. Lupeol, a triterpene found in the fraction (F8) eluted by 10% ethyl acetate/90% hexane and accounted for 19.3% (*w*/*w*) of F8, inhibited the proliferation of PC3 cells. Both lupeol and F8 induced G2/M arrest, inhibition of β-catenin signaling, regulation of apoptotic signal molecules (cytochrome c, Bcl-2, P53, and caspase 3 and 8), and suppression DNA repair enzyme expression (Uracil-DNA glycosylase (UNG)). To our knowledge, our study is the first report that lupeol inhibited the expression of UNG to elicit the cytotoxicity against androgen-insensitive-prostate-cancer cells. Collectively, Luobuma, which contains several antitumor bioactive compounds, holds the potential to be a dietary chemopreventive agent for prostate cancer.

## 1. Introduction

Luobuma (known as dogbane, Indian hemp, or *Apocynum venetum* L. (AVL)), which is found in South Europe, North Africa, and Asia, is a commonly consumed tea beverage, and has been linked to the ameliorative effects on depression, anxiety, hypertension, and cardiovascular diseases [1]. Several bioactivities of the leaf extracts have been revealed, which include cardiotonic [2], diuretic [3], antioxidative [4], and antihypertensive [5] activities. Constituents such as organic acids, phloroglucinols, phytosterols, glycosides, triterpenoids, and polyphenols [1] that have been identified in leaves and flowers may contribute to these bioactivities and make Luobuma tea a potential cancer preventive agent [6]. However, there is scant evidence to indicate if AVL can be a potential chemopreventive agent against androgen-independent prostate cancer (AIPC).

Prostate cancer (PC) is an aging disease. Autopsy studies indicated that almost 50% of males over the age of 50 may experience prostate cancer [7]. Most prostate cancers are androgen-dependent (with androgen receptor (AR) expression) adenocarcinomas, which possess a glandular formation, and can produce prostate-specific antigen (PSA) under the regulation of AR activity. The progression of PC is induced by the binding of dihydrotestosterone (DHT) to AR. DHT is formed by the reduction of testosterone that is catalyzed by 5-α-reductase. This androgen hormone that binds to AR will be translocated subsequently to the nucleus to trigger anti-apoptotic effects [8]. The ablation of androgen is the common treatment for PC. However, the progression of androgen-independent, castration-resistant prostate cancer (CRPC) may occur 1–3 years after initial androgen ablation surgery. These androgen-independent and PSA-free cancer cells, such as prostatic, small cell neuroendocrine carcinomas (SCNC), are aggressive, metastatic adenocarcinomas [9].

Wnt/β-catenin signaling pathway plays a critical role in CRPC recurrence and resistance. This signaling pathway undergoes significant genomic alterations, and more β-catenin nuclear translocalization and co-localization with AR in CRPC compared to hormone naïve primary prostate cancer, which shows no alterations and less translocation [1]. Wnt/β-catenin signaling pathway involves in cell–cell adhesion and cell proliferation. When “Wnt off”, cytopalsmic β-catenin is complexed with APC, GSK-3β, β-TrCP, and axin/conductin, and degraded by phosphorylation at Ser/Thr residues by GSK-3β [10,11]. When “Wnt on”, the activation of Wnt pathway suppresses the activity of GSK-3β allowing the subsequent nuclear translocation of β-catenin, which forms complex with TCF/LEF family to transcribe Wnt target genes for cell proliferation [12]. Elevated β-catenin level is usually associated with prostate tumor growth [13]. Thus, the regulation of the Wnt/β-catenin pathway can be a therapeutic approach for CRPC.

The conventional cancer treatments aim at damaging the DNA of cancer cells, which subsequently halts cancer cell proliferation. Although those strategies may demonstrate high efficiency on tumors at an early stage, they suffer therapeutic resistance owing to drug-resistance or cell mutations [14]. The synergistic cytotoxic effect that is induced by combinatory therapy may improve therapeutic efficacy of conventional treatment through sensitizing cancer cells to DNA-damaging agents by downregulation of DNA-repair pathways. For example, uracilation of DNA commonly indicates a sign of DNA mutation (from G:C to A:T) that is caused by cytosine deamination or dUTP misincorporation. It is reported that activation of base excision repair (BER) pathway for DNA repair was initiated by uracil DNA glycosylase (UNG) [15]. The loss of UNG has been shown to be associated with the induction of apoptosis in human prostate cancer cells and display of increased sensitivity to genotoxic stress [14]. Therefore, the disruption of UNG may be a valuable strategy, in combination with DNA-damaging agents, for synergistic cancer therapy.

In this study, we aimed to explore the major bioactive compounds of ethanolic extracts of AVL that act against castration-resistant prostate cancer such as SCNC, and to investigate the associated molecular cytotoxic mechanisms. We performed activity-guided fractionation of ethanolic extracts, and we isolated/characterized the bioactive compounds by High Performance Liquid Chromatography (HPLC), Liquid Chromatography-Mass Spectrometry (LC-MS), and Nuclear Magnetic Resonance (NMR). The properties of selected fractions, such as antioxidative activity, total content of phenolics, flavonoids and terpenoids, were characterized to facilitate the identification of bioactive compounds. PC3 cells, an AIPC cell line that shares similar cytokeratin profiles and neuroendocrine (NE) markers with SCNC [9], was used as a test model in vitro. Apoptotic signaling and UNG activity were also examined. Our findings suggested that ethanolic extracts of AVL leaves exerted multifunctional anticancer activities on PC3 cells. AVL extracts induced G2/M arrest, inhibition of β-catenin signaling, regulation of apoptotic signal molecules (cytochrome c, Bcl-2, P53, and caspase 3 and 8), and suppression of DNA repair enzyme expression (Uracil-DNA glycosylase, UNG). Lupeol, a major bioactive compound of AVL extracts, inhibited the activity of UNG, which sensitized prostate cancer cells to cytotoxic stress induced by ethanolic extracts of AVL leaves. Taken together, Luobuma (AVL) holds the potential to be a promising chemopreventive agent against androgen-insensitive prostate cancer.

## 2. Materials and Methods 

### 2.1. Chemicals

Lupeol, kaempferol, and β-sitosterol were purchased from Sigma-Aldrich (St. Louis, MO, USA). Isorhamnetin and quercetin were obtained from the United States Pharmacopeial Convention (Rockville, MD, USA). Stigmasterol was acquired from Santa Cruz Biotechnology (Dallas, TX, USA). All chemicals and reagents, namely methanol, *n*-hexane, ethanol, and ethyl acetate, were of analytical or HPLC grade unless otherwise specified.

### 2.2. Extraction

**Figure 1:**
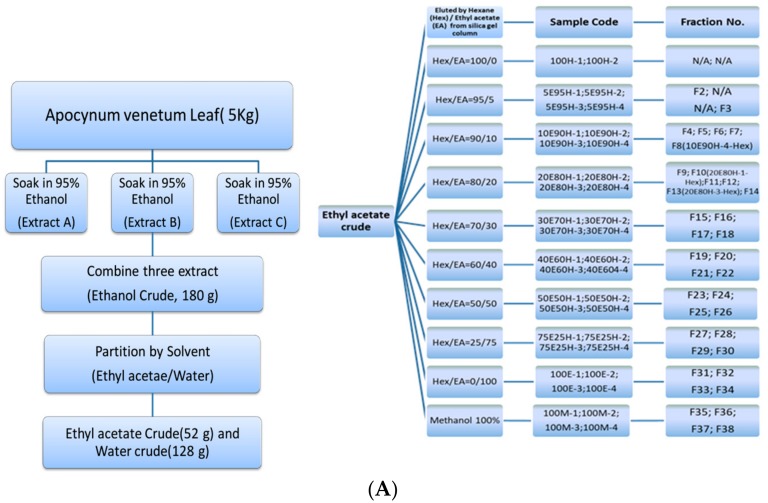

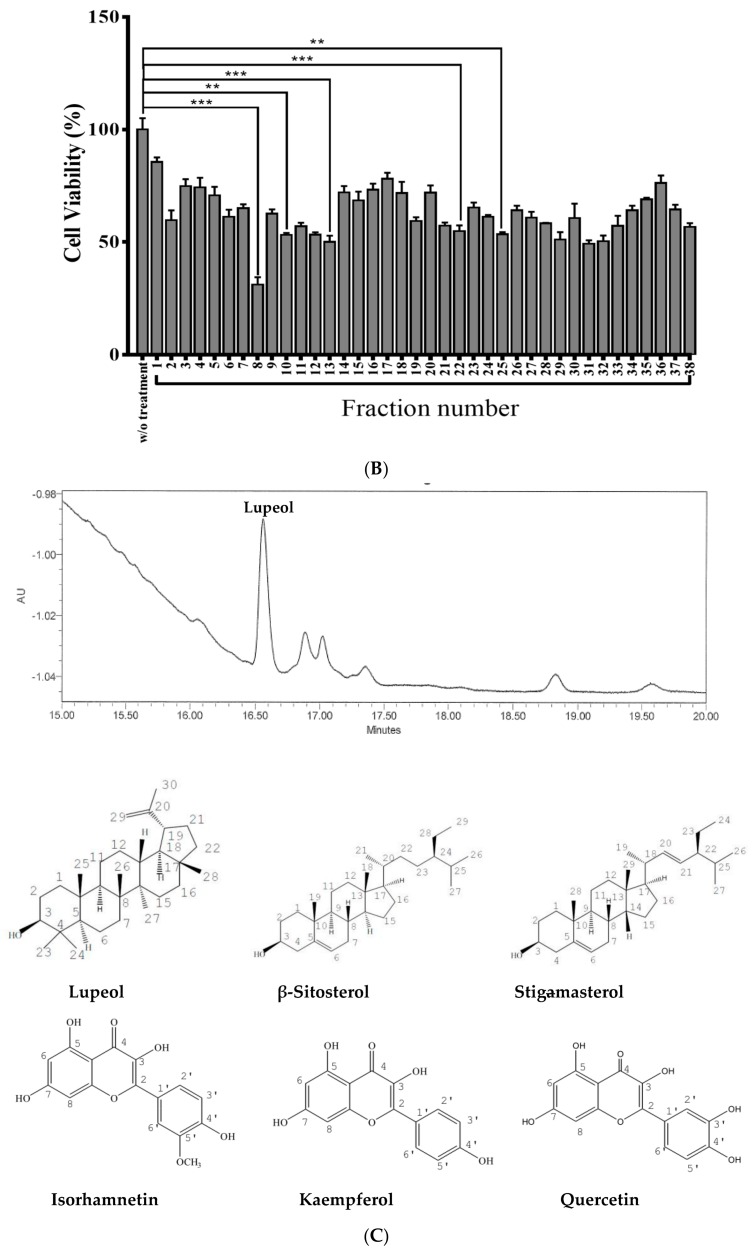
Isolation and cytotoxicity of *Apocynum venetum* L. (AVL) ethanolic extracts: (**A**) Flow chart of the AVL extraction process (H: hexane; E: ethyl acetate; M: methanol; N/A: not applicable); (**B**) The cytotoxicity of fractions on PC3 cell viability (250 μg/mL). Cell viability was determined by MTT (3-(4,5-cimethylthiazol-2-yl)-2,5-diphenyl tetrazolium bromide) assay (3 × 10^4^ cells/well, incubated with Ham’s F-12K medium with 10% Fetal Bovine Serum, (FBS). MTT assay was detected at 570 nm; (**C**) Chromatogram of 10E90H-4 and chemical structures of isolated phytocompounds from AVL leaves ethanolic extracts. The absorbance was recorded at 210 nm. All data are expressed as the means ± standard deviation (SD) of at least three replicates (*n* ≥ 3). ** *p* < 0.05; *** *p* < 0.01.

Briefly, the dried leaves of AVL were purchased locally, and ground fine to promote its homogeneity (Figure 1A). The extract of fine ground powder was soaked in 95% (*v/v*) alcohol with continuous stirring (100 rpm) for 1 h before filtration (approximately 5 kg of fine ground powder in 15 L of ethanol). The filtrate was concentrated using a rotary evaporator under reduced pressure (12.6 psi). The above procedure was repeated twice, and the collected ethanolic extracts from each iteration were combined (180 g) before fractionation. The ethanolic extracts were further liquid–liquid partitioned by ethyl acetate (52 g) and water (128 g). The ethyl acetate crude extracts were fractionated further on a silica gel column, eluting with hexane (H), ethyl acetate (EA), and methanol (M). A total of 38 fractions of EA crude extracts were obtained, and the solvent was evaporated to dryness under a stream of nitrogen at 45 °C for long-term storage. The effective compounds in different fractions were analyzed and identified by LC-MS, NMR, and UPLC.

### 2.3. LC-MS Analysis 

Effective compounds in different fractions were identified using liquid chromatography positive mode–electrospray ionization–mass spectrometry LC-ESI-MS, Applied Biosystems, API-4000™ (Foster City, CA, USA) with the Q1 scan mode by infusing individual samples that were prepared in methanol. The parameters of ESI-MS were optimized with fragmentor voltage 120 V, capillary voltage of 3000 V, drying gas temperature of 550 °C, drying gas flow rate of 5 L/min, nebulizer pressure of 50 psi, and auxiliary gas at 50 psi.

### 2.4. NMR Analysis

Six candidate compounds were analyzed and identified by elucidating ^1^H- and ^13^C-nuclear magnetic resonance spectra Bruker AVANCE II 400 NMR spectrometer (Indianapolis, IN, USA) in CDCl_3_. The chemical shifts were in ppm and coupling constants were in Hz.

### 2.5. UPLC Analysis

The chromatographic separation was performed using a Waters ACQUITY UPLC system with a PDA e-λ detector (Manchester, UK) and a Waters ACQUITY UPLC HSS T3 Column (100 Å, 1.8 μm, 2.1 mm × 50 mm). The binary mobile phase consisted of 0.2% acetate acid in water (A) and acetonitrile (B). The flow rate was controlled precisely by a binary solvent pump at 0.4 mL/min. Linear gradients from 10%B/90%A to 100%B/0%A over 20 min were used (the linear gradient sequence was 0–8 min: 10% B; 8–10 min: 40–80% B; 10–16 min: 80–100% B; 16–20 min: 100% B). The injection volume of the sample was 2 μL (partial loop with Needle overfill) and the absorbance was recorded at 210 and 254 nm.

### 2.6. The Total Phenolic, Flavonoid, Triterpene Contents and Antioxidant Properties

Total phenolic content (TPC) of extracts was carried out according to the Folin–Ciocalteu method [16]. Gallic acid (0.352, 0.704, 1.04, 1.44 μmol/mL) was used for the construction of a calibration curve. All tests were carried out in triplicate. TPC was calculated from the calibration curve, and results were expressed as gallic acid equivalents in 1 g dry extract. Total flavonoid content (TFC) of extracts was determined as in Liu et al. [17]. Catechin (0.012, 0.024, 0.036, 0.048 μmol/mL) was used for the construction of a standard curve. All tests were done in triplicate, and the flavonoid content of extracts was reported as milligram of catechin equivalents per 1 g dry extract [17].

The triterpene content was obtained by previously reported chromogenic method by Liu et al. [18] with modification, and expressed as oleanolic acid (%). Briefly, an aliquot of sample solution (200 μL) was added and evaporated to dryness in a water-bath, which was followed by the addition of 0.1 mL 5% (*w/v*) vanillin-acetic solution and 0.8 mL sulfuric acid and incubation at 65 °C for 25 min. After cooling down to room temperature, the mixture was diluted to 10 mL with acetic acid and measured at 545 nm against blank (containing all reagents except sample solution) using a spectrophotometer (Agilent/HP 8453, Santa Clara, CA, USA). The content was determined using the standard oleanolic acid calibration curve.

DPPH (1,1-diphenyl-2-picrylhydrazyl) radical-scavenging activity was determined as described by Wu et al. [16]. Ascorbic acid was chosen as the reference standard compound and all tests were done in triplicate. DPPH radical scavenging ability of the test sample was expressed as IC_50_, the amount of tested extract (mg) required for a 50% decrease in absorbance of DPPH^•^, expressed in terms of ascorbic acid equivalents. The inhibition percentage for scavenging DPPH^•^ was calculated according to the equation (A_control_ − A_test_/A_control_) × 100%, where A_control_ is the absorbance of the control (containing only DPPH^•^ solution) and A_test_ is the absorbance of the test sample. The absorbance (515 nm) of DPPH^•^ was plotted against the antioxidant concentrations as standard curves to calculate IC_50_.

ABTS^•+^ (2,2′-azinobis-(3-ethylbenzothiazoline-6-sulfonic acid) radical cation) assay was based on the modification of the method reported by Wu et al. [16]. Trolox, a vitamin E analog, was selected to serve as a standard. The inhibition (%) of ABTS^•+^ absorbance was calculated based on the expression (A_control_ − A_test_/A_control_) × 100%. The absorbance (734 nm) of ABTS^•+^ was plotted against the antioxidant concentrations as standard curves to calculate IC_50_. Results of the assay are expressed in terms of TEAC (Trolox equivalent antioxidant capacity).

### 2.7. Cell Culture and Proliferation Assay 

Human PCa cells (3 × 10^4^ cells/well, 12-well-plate), PC3 cells, which were maintained at 37 °C and 5% CO_2_ humidified atmosphere in F-12 Ham Kaighn’s Modification (Ham’s F-12K) that contained 10% fetal bovine serum (FBS) and 1% penicillin–streptomycin solution, were used in all experiments. Cell viability was determined by MTT assay [16]. Aliquots (20 μL/well) of MTT (3-(4,5-Dimethylthiazol-2-yl)-2,5-diphenyltetrazolium bromide) stock solution were introduced. Cell medium was then aspired after 4 h of incubation, followed by the addition of dimethyl sulfoxide (DMSO) (100 μL) to dissolve formazan for another 10 min. Absorbance at 570 nm was measured to determine cell viability. Results are revealed from three replicate runs.

### 2.8. Cell Cycle Analysis 

Cells were fixed in ice-cold 75% ethanol at 4 °C for 30 min, followed by several washes with phosphate-buffered saline (PBS). After incubation (30 min) with 100 μg/mL RNase A and 40 μg/mL propidium iodide (PI) at 37 °C, cells were subjected to flow cytometric analysis (Becton Dickinson, Mountain View, CA, USA). Cell cycle phases were analyzed and calculated by CELLQuest software (version 5.1, San Jose, CA, USA).

### 2.9. Assay of Caspase Activity

Caspase activity (caspase 2, 3, 8, 9, and 12) was determined using a Caspase fluorometric kit (R&D Systems, Minneapolis, MN, USA). All procedures were based on the manufacturer’s instructions.

### 2.10. Western Blot Analysis

Cells were lysed at 4 °C in RIPA (radioimmunoprecipitation assay) buffer that contained 50 mM Tris, pH 7.4; 150 mM NaCl; 1% Triton X-100 (Sigma, St. Louis, MO, USA); 0.25% sodium deoxycholate; 5 mM EDTA, pH 8.0; 1 mM EGTA, pH 8.0), and supplemented with 0.5 mM DTT, 5 μg/μL leupeptin, 0.2 μM PMSF, 5 μg/μL aprotin, 1 μM Na_2_VO_4_, and 1 μM NaF. Total protein concentration was measured by the Bradford method. Proteins were separated by SDS-PAGE followed by electrotransfer onto a polyvinylidene difluoride (PVDF) membrane. Primary antibodies of Bcl-2, cytochrome c, caspase 3, caspase 8, pP53, UNG, β-catenin, and β-actin were applied overnight (4 °C) after 1 h of blocking with PBS that contained 0.1% Tween-20 and 10% non-fat dry milk (NFDM). Secondary HRP-conjugated antibodies (1:10,000) were co-incubated with a membrane (1 h) after extensive washes with PBST (phosphate buffer saline with Tween-20). The immunoreactive blots were visualized using a Western Lightning Chemiluminescence kit (Perkin Elmer LAS, Boston, MA, USA).

### 2.11. Statistical Analysis

All data are expressed as the means ± standard deviation (SD) of at least three replicates (*n* ≥ 3). Student’s *t* test was performed for evaluation of statistical significance.

## 3. Results

### 3.1. Extraction, Fractionation, and Cytotoxicity Induced by AVL Ethanolic Extracts

A total of 38 fractions were obtained (Figure 1A). The cytotoxic effect of AVL ethanolic extracts on cell viability of PC3 cells was determined by a MTT assay (Figure 1B). PC3 cells were treated with various fractions (fractions No. 1–38 (F1–F38), 250 μg/mL)) for 24 h. Our results showed that fractions No. 8, No. 10, No. 13, No. 22, No. 25, No. 29, No. 31 and No. 32 significantly (*p* < 0.01) induced cell death with survival rates of 27.14 ± 0.22%, 53.11 ± 0.86%, 50.10 ± 2.70%, 54.84 ± 2.51%, 53.43 ± 0.96%, 51.11 ± 3.31%, 49.20 ± 1.51% and 50.29 ± 2.56%, respectively. Among all fractions, fraction No. 8 (F8) exhibited the most significant cytotoxicity. Based on the extraction efficiency and cytotoxcicity, fractions No. 8, No. 10, No. 13, No. 22, and No. 25 were selected for further characterization.

### 3.2. Identification and Analysis of Bioactive Compounds

It was suggested that malignancy of PC can be promoted by elevated oxidative stress. Several antioxidative bioactive compounds such as polyphenolic compounds, flavonoids, and triterpenes demonstrate anti-prostate cancer activity. The measurement of antioxidative activity and total polyphenol, total flavonoid, and total triterpene contents of the cytotoxic fractions (No. 8, No. 10, No. 13, No. 22, and No. 25) were then performed (Table 1). Results indicated that, in addition to the possession of a strong cytotoxic activity, F8 contained the highest amount of flavonoids and a moderate amount of triterpene and phenolic compounds among the studied fractions. Moreover, F8 also exhibited the highest antioxidative ability (confirmed by ABTS and DPPH assays) among the studied fractions. Based on the strong cytotoxic effect and the high antioxidative activity on PC3 tumor cells, F8 (10% ethyl acetate/90% hexane; 10E90H-4) was further examined by NMR, mass spectroscopy, and UPLC.

^1^H NMR spectral analysis of isolated F8 peaks indicated that there were six tertiary methyl groups (C23~C28) in the region of δ 0.75–1.68, and a vinylic methyl attached to C-30. An H-3 axial portion appeared at δ 3.20 as a multiplet peak. H-19 appeared as a multiplet at δ 2.38. Moreover, C-29 methylene protons appeared as broad singlet peaks at δ 4.56 and 4.68. After a comparison of these spectra with published reports [19] and after performing MS analysis (positive Q1 scan mode) of a commercial standard (*m*/*z* 449 [M + Na]^+^, 409 [M − H_2_O]^+^), we confirmed that lupeol was one of the constituents in F8. UPLC quantitative analysis revealed that lupeol (chromatogram was shown in Figure 1C) was about 19% (*w*/*w*) of F8. Other bioactive components in different fractions were also analyzed and identified by NMR, mass spectroscopy, and UPLC. We identified β-sitosterol, sitgmasterol, isorhamnetin, kaempferol, and quercetin (chemical structures shown in Figure 1C) in different fractions of AVL ethanolic extracts. The UPLC quantitative analysis and NMR, MS spectra of identified constituents are listed in Table 2.

### 3.3. AVL Fraction No. 8 Inhibited Cell Proliferation by G2/M Arrest in PC3 Cells

**Figure 2:**
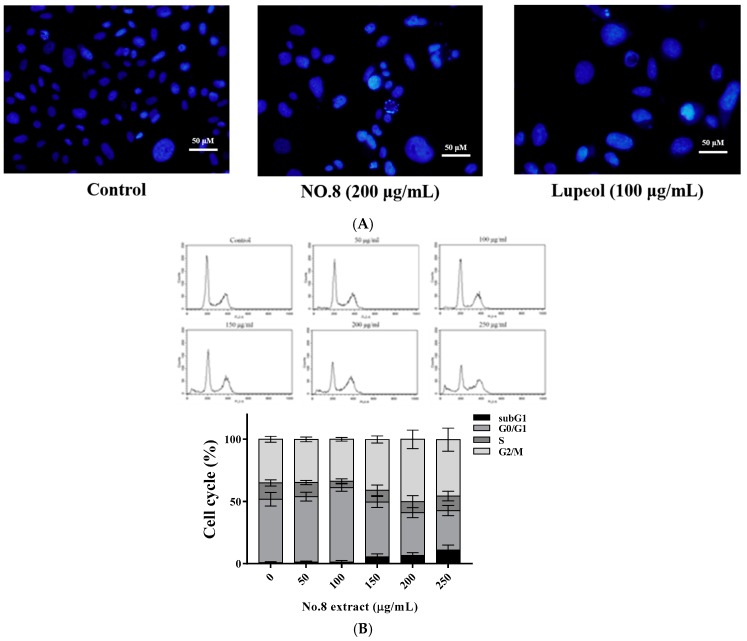

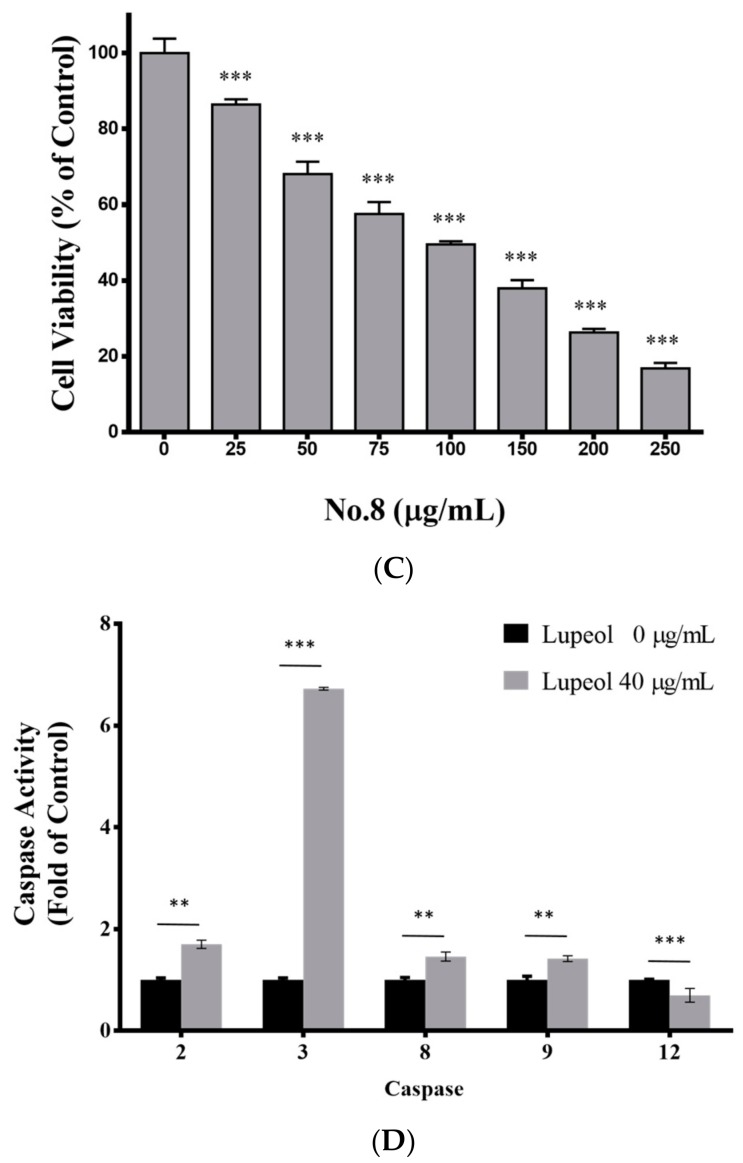
Apoptotic effect induced by AVL (*Apocynum venetum* L.) F8 and lupeol on PC3 cells. (**A**) DAPI (4′,6-diamidino-2-phenylindole) staining of AVL No. 8 or lupeol-treated PC3 cells; (**B**) flow cytometric analysis of propidium iodide staining was used to analyze apoptosis in PC3 cells following vehicle-treatment and 50, 100 μg/mL for 24 h; (**C**) PC3 cells were treated with F8 at designated concentrations for 24 h. Cell viability was determined by MTT (3-(4,5-Dimethylthiazol-2-yl)-2,5-diphenyltetrazolium bromide) assay; and (**D**) lupeol-induced caspase activation in PC-3 cells. All data are expressed as the means ± standard deviation (SD) of at least three replicates (*n* ≥ 3). Statistical significance was calculated by comparing with control group. ** *p* < 0.05; *** *p* < 0.01.

**Figure 3:**
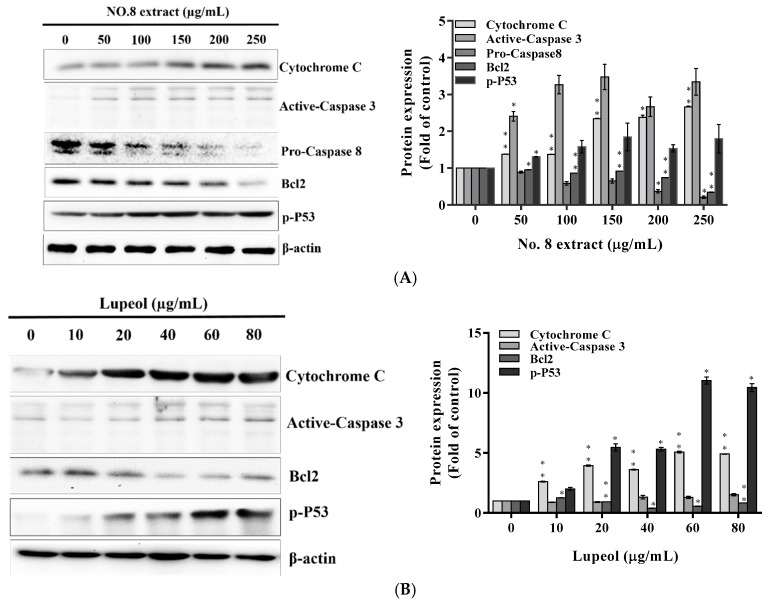
Regulation of apoptotic molecules by AVL (*Apocynum venetum* L.) F8 on PC3 cells. The apoptotic molecules were analyzed by Western blot after PC3 cells were treated by: F8 (**A**); or lupeol (**B**). β-actin was used as an internal standard. All data are expressed as the means ± standard deviation (SD) of at least three replicates (*n* ≥ 3). Statistical significance was calculated by comparing with control group. * *p* < 0.05; ** *p* < 0.01.

To investigate mechanisms of cell death in PC3 prostate carcinoma cells induced by F8, DAPI (4′,6-diamidino-2-phenylindole) staining and cell cycle analysis were carried out. PC3 cells demonstrated nuclear condensation (Figure 2A) and a subG1 phase increase to 7.62% (Figure 2B) after co-incubation with 200 μg/mL of F8 for 24 h. The cell cycle profile also (F8: 0, 50, 100, 150, 200, and 250 μg/mL) revealed that F8 (200 μg/mL) markedly induced an increase in G2/M. The percentage of G2/M phase increased initially from 32.6% to approximately 41.7% (Figure 2B). Additionally, as indicated in Figure 2C, F8 dose-dependently (0, 25, 50, 75, 100, 150, 200, and 250 μg/mL) elicited cytotoxicity and inhibited PC3 cell proliferation with an IC_50_ value of 99.02 μg/mL (Figure 2C). We inferred from these findings that lupeol within F8 (Table 2A) could induce apoptosis (Figure 2A and Figure 3B) and activate apoptotic caspases (Caspase 3, 8, and 9; Figure 2D).

It has been indicated that lupeol activated apoptotic molecular machinery in AR-positive LNCaP cells [20,21]. To further explore whether lupeol might be a compound responsible for the apoptotic effect of AVL F8 against AR-negative PC3 cells, apoptotic molecules were then determined. As expected, increased levels of p-P53 in both treatments of F8 and lupeol triggered intrinsic apoptosis through the release of cytochrome c and downregulation of Bcl_2_, followed by the subsequent activation of caspase 3 (Figure 3A,B). Additionally, the downregulation of procaspase 8 of F8 implied the elicitation of extrinsic apoptosis (Figure 3A). Collectively, the molecular (Figure 2 and Figure 3) and chemical analyses (Figure 1) evidence implied that lupeol was a key bioactive compound in F8 that induced cytotoxicity against PC3 cells.

### 3.4. AVL F8 Impaired DNA Repair System through Downregulation of the Expression of Uracil-DNA Glycosylase Leading to the Promotion of G2/M Arrest

**Figure 4:**
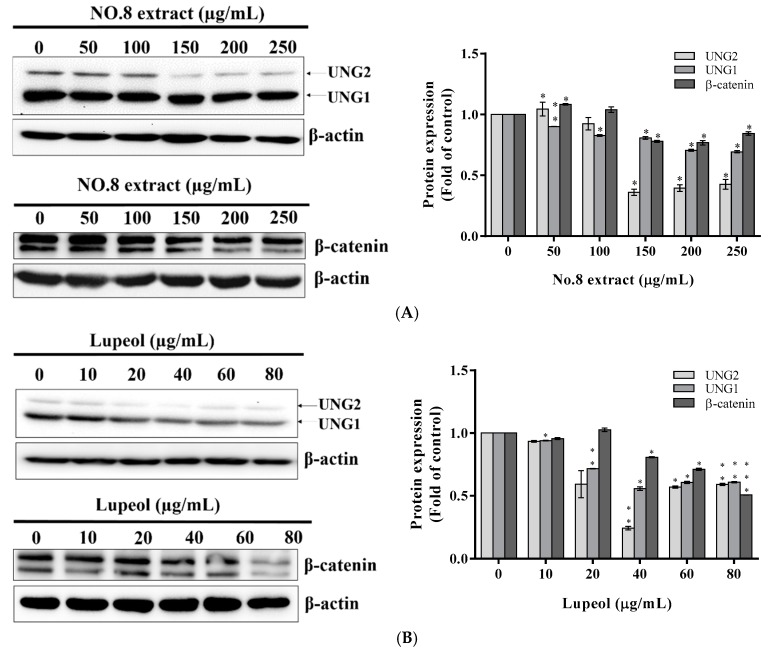
Lupeol and AVL (*Apocynum venetum* L.) F8-inhibited expression of UNG (uracil-DNA glycosylase) and the down-regulated β-catenin signaling pathway in PC-3 cells. The UNG and β-catenin molecules were analyzed by Western blot after PC3 cells were treated by: F8 (**A**); or lupeol (**B**). β-actin was used as an internal standard. All data are expressed as the means ± standard deviation (SD) of at least three replicates (*n* ≥ 3). Statistical significance was calculated by comparing with control group. * *p* < 0.05; ** *p* < 0.01; *** *p* < 0.001.

Apoptosis is a highly regulated cellular process, and the progression of apoptosis results from a disturbed cell cycle. Lupeol provoked apoptosis at caspase 3-regulated G2/M phase [21], which was potentially induced by an impaired DNA repair system. Uracil-DNA glycosylase (UNG) is an essential DNA repair enzyme. Depletion of UNG would result in DNA damage and induce apoptosis. Therefore, the inhibitory effect of lupeol and F8 on the expression of UNG was investigated. PC3 cells were co-incubated with lupeol (0, 10, 20, 40, 60, and 80 μg/mL) and F8 (0, 50, 100, 150, 200, and 250 μg/mL) for 24 h. Immunoblotting analysis revealed that lupeol and F8 restrained the expression of UNG significantly in a dose-dependent manner (Figure 4A,B). These findings suggested that F8 and lupeol exerted multipronged approaches to suppress PC3 cell proliferation. AVL F8 and lupeol could be a good candidate for prostate cancer synergistic therapy while using DNA-damaging drugs due to the alteration of UNG expression.

### 3.5. Reduced β-Catenin Expression Involved in AVL F8-Induced G2/M Arrest

The Wnt/β-catenin pathway plays a part in cell proliferation and differentiation in prostate cancer. A decrease in β-catenin levels induced cell cycle arrest at G2/M in pancreatic adenocarcinoma [22] and epidermal keratinocytes [23]. Accordingly, the activity of β-catenin was examined by co-incubation of PC3 cells with F8 (0, 50, 100, 150, 200, and 250 μg/mL for 24 h) and with lupeol (0, 10, 100, 20, 40, 60, and 80 μg/mL for 24 h). The expression of β-catenin was dose-dependently downregulated by F8 and lupeol (Figure 4A,B), which also induced a G2/M arrest of PC3 cells, as observed in Figure 2B. These findings suggested that lupeol serves as a key role in β-catenin suppression, thereby leading to G2/M arrest.

## 4. Discussion

Luobuma tea, prepared from the leaves of *Apocynum venetum* L., is a popular tea beverage in Asia, which has long been suggested empirically to be associated with longevity, and it has been reported to possess multiple bioactivities. The bioactive compounds responsible for the corresponding pharmacological activity have been addressed [1]. However, there is insufficient evidence to indicate that this herbal remedy possesses anti-prostate cancer activity, especially for androgen-insensitive prostate cancer. The main purpose of this work was to identify the major bioactive compounds through the execution of activity-guided fractionation of the ethanolic extracts that showed cytotoxic effect toward PC3 cells. In addition, the molecular mechanisms behind the cytotoxic activity were also investigated.

Initially, we inferred that the effective anticancer constituent of F8 was credited to the presence of flavonoids. Interestingly, the identification of lupeol, a major compound that accounted for approximately one-fifth (19.3% *w*/*w*) (Table 2) of F8, revised our hypothesis. The finding of this dietary triterpene in F8 partially explained the effectiveness of the anticancer activity. Lupeol has been reported to be a potent anticancer agent against androgen-dependent prostate cancer, such as LNCaP [24], and androgen-independent prostate cancer, such as PC3 cells [21]. We examined further if lupeol was responsible for the anticancer activity of F8 on androgen-independent prostate cancer. The anticancer molecular mechanisms of lupeol and F8 then underwent parallel investigation. Results indicated that lupeol and F8 elicited similar anti-proliferative mechanisms including the induction of G2/M arrest, apoptosis, and suppressed expression of UNG and β-catenin. We thus confirmed that lupeol was one of the major anticancer compounds contained in F8.

Previous studies demonstrated that lupeol was an effective and multi-target anticancer agent. Lupeol caused elicitation of apoptosis through G2/M arrest (PC3 cells) [21] and reduction of β-catenin signaling [13] in AR-negative human prostate cancer (DU145 cells). Additionally, lupeol inhibited the growth of androgen-dependent prostate cancer, LNCaP cells [24]. These observations were in accordance with our finding that both lupeol and F8 suppressed β-catenin signaling, inducing G2/M arrest and activating subsequent apoptosis in PC3 cells. We thus speculated that F8 may inhibit the growth of androgen-dependent prostate cancer cells due to the existence of lupeol. Moreover, lupeol was able to enter cellular membranes to form stomatocytes in red blood cells [25] due to its amphiphilic feature, contributed by a single hydroxyl group and a large apolar skeleton. This feature could make lupeol penetrate cancer cells and exert anti-cancer activity.

The crosstalk between β-catenin signal and DNA repair machinery found in the present study may propose a new cytotoxic mechanism of lupeol. The suppression of β-catenin could elicit the generation of reactive oxygen species and trigger oxidative stress [26]. Damaged DNA caused by oxidative stress promoted the signaling of p53-induced phosphatase for base excision repair [27]. PPM_1_D (protein phosphatase 1D), a p53-activated phosphatase, further suppressed base excision repair through hindering UNG from activation [28] leading to the progression of apoptosis. Furthermore, thymine DNA glycosylase (TDG), a member of DNA glycosylase superfamily, positively regulated the expression of β-catenin [29]. Despite their different mechanisms in DNA damage search, recognition, and excision, DNA glycosylases may share common activities [30]. The reduction in UNG observed in our study would possibly promote the downregulation of β-catenin expression. These mechanisms need to be further confirmed. In the present study, though the reduced UNG protein level was determined, the activity of UNG (such as DNA damage, double-strand break (DSB) formation, and DSB repair capacity) has to be investigated for better implication of uracil DNA glycosylation under the effect of lupeol.

Lupeol is suggested to be applied in combination with microtubule agent for combinatory therapy because of its ability in induction of G2/M arrest, which is regulated by microtubule assembly [31]. Combinatory therapy has the merits of reduced drug resistance, low failure rate, fewer side-effect than monotherapy [32]. Phytochemicals that exert diversified interactions can be used as multi-target drugs for disease treatments. The bioactive components contained in AVL may function synergistically to achieve the eradication of prostate cancer cells. Flavonoids, such as quercetin, may elicit DNA damage [33] and cause cytotoxicity, which was augmented by lupeol through its suppressive effect on DNA repairing. Similar synergistic cytotoxic effect was observed in quercetin and hyperoside on androgen-dependent (LNCaP) and androgen-independent (PC3) prostate cancer [34].

In addition to flavonoids, triterpenoids can also induce DNA damage. Ganoderic acid DM (GADM) and a synthetic triterpenoid, CDDO (2-cyano-3,12-dioxooleana-1,9(11)-dien-28-oic acid)-imidazolide, caused DNA damage and triggered G1 [35] and G2/M arrest [36] in human breast cancer, respectively. GADM also demonstrated anti-proliferative effects on both androgen-dependent and androgen-independent prostate cancer cell lines [37]. Although the phytosterols that we identified in AVL, stigamasterol and β-sitosterol, showed preventative effect on DNA damaging, these two plant sterols exhibited anticancer activity [38]. Stigmasterol inhibited the growth of skin carcinoma through elevated levels of antioxidative enzymes (glutathione, catalase, and superoxide dismutase) and reduced lipid oxidation and DNA damage [39]. β-Sitosterol evoked apoptosis in prostate cancer LNCap cells through the production of ceramide [40]. Taken together, the phytochemicals in *Apocynum venetum* L. may act as multitargeting anticancer agents or function synergistically to suppress tumor growth.

## 5. Conclusions

There is scant evidence regarding the antitumor activity of *Apocynum venetum* L. (AVL), although its ameliorative effects have been suggested elsewhere. In the present study, we isolated and identified anticancer bioactive compounds from the ethanolic extracts of AVL leaves by UPLC, LC-MS, and NMR. We also verified the existence of polyphenols, phytosterol, and triterpenoid in F8, which were potential anticancer compounds. Lupeol was one of the abundant bioactive compounds that demonstrated a significant anti-proliferative effect on PC3 cells. Lupeol not only exerted its pro-apoptotic activity against cancer cells, but also could synergistically function with DNA-damaging natural compounds to augment cell cytotoxicity through disturbing DNA repairing systems. In addition, lupeol downregulated β-catenin signal during G2/M phase, and sensitized prostate cancer cells to cytotoxic stress of AVL extracts by the suppression of UNG activity. In summary, lupeol and other anticancer agents (e.g., polyphenols and phytosterols) found in the ethanolic extracts of *Apocynum venetum* L. exerted their cytotoxicity by different mechanisms that make Luobuma tea a potential chemopreventive agent against androgen-insensitive prostate cancer.

## Figures and Tables

**Table 1:** The characteristics of AVL ethanolic fractions on antioxidative activity, and the total content of flavonoids, polyphenols, and triterpenoids.

Fraction	IC_50_ (24 h)	Total Flavonoid Content	Total Phenolic Content	Total Triterpenoids	ABTS^+^	DPPH
	μg/mL	mg Catecin Equivalents/g	mg Gallic Acid Equivalents/g	Oleanolic Acid (%)	μmol Trolox Equivalents/g	μmol Vitamin C Equivalents/g
**No. 8**	99	100.3 ± 4.9	28.3 ± 1.2	51.6 ± 6.3	65.2 ± 1.0	34.6 ± 0.2
**No. 10**	216	64.0 ± 5.5	35.5 ± 0.1	49.4 ± 2.4	180.9 ± 3.0	55.1 ± 0.1
**No. 13**	274	25.2 ± 0.8	12.8 ± 0.2	26.3 ± 1.6	118.2 ± 1.6	77.4 ± 0.4
**No. 22**	240	64.0 ± 0.4	54.8 ± 0.1	79.4 ± 7.5	196.1 ± 2.7	80.4 ± 0.4
**No. 25**	227	86.8 ± 0.5	51.9 ± 1.2	39.4 ± 3.0	251.6 ± 3.6	96.5 ± 1.4

**Table:** (**A**)

Compounds	In AVL *	In Fract. ^#^	In AVL *	In Fract. ^#^	In AVL *	In Fract. ^#^	In AVL *	In Fract. ^#^	In AVL *	In Fract. ^#^
Fraction	No. 8		No. 10		No. 13		No. 22		No. 25	
***Sterols***										
Sitgmasterol	5.01	4.09	10.71	25.10	0.88	1.34				
β-sitosterol			1.66	3.89	19.10	29.07				
***Triterpenoid***										
Lupeol	23.65	19.31	1.25	2.92						
***Flavonoids***										
Kaempferol							0.37	0.60	0.12	0.36
Isorhamnetin									0.05	0.16

* Yield in *Apocynum venetum* L. AVL (mg/kg AVL); **^#^** Yield in fraction (%).

**Table:** (**B**)

Compounds	Molecular Formula	Molecular Weight	Q1 Mass (*m*/*z*)
***Sterols***			
Sitgmasterol	C_29_H_48_O	412.69	413 [M + H]^+^
β-sitosterol	C_29_H_50_O	414.71	437 [M + Na]^+^
***Triterpenoid***			
Lupeol	C_30_H_50_O	426.72	449 [M + Na]^+^
***Flavonoids***			
Kaempferol	C_15_H_10_O_6_	286.23	287 [M + H]^+^
Isorhamnetin	C_16_H_12_O_7_	316.26	317 [M + H]^+^
Quercetin	C_15_H_10_O_7_	302.24	303 [M + H]^+^

**Table:** (**C**)

Compounds	Selected 1H-NMR Data δ (Multiplicity/Hz)	Selected 13C-NMR Data δ
***Sterols***		
Sitgmasterl	0.70 (3H, s, H-29), 0.82(3H, d, H-26), 0.84 (3H, d, H-24), 0.85 (3H, d, H-27), 1.03 (3H, s, H-28), 3.53 (1H, m, H-3), 5.02 (1H, dd, H-21), 5.16 (1H, dd, H-20), 5.35 (1H, d, H-6)	12.0 (CH3, C-18), 12.3 (CH3, C-29), 18.8 (CH3, C-21), 19.0 (CH3, C-27), 19.4 (CH3, C-19), 21.0 (CH3, C-26), 21.1 (CH2, C-11), 24.4 (CH2, C-15), 25.4 (CH2, C-23) 28.9 (CH, C-25), 31.6 (CH2, C-2), 31.9 (CH, C-8), 31.9 (CH2, C-7), 36.5 (C, C-10), 37.2 (CH2, C-1), 39.7 (CH2, C-12), 42.2 (CH2, C-4), 42.3 (C, C-13), 50.1 (CH, C-9), 56.0 (CH, C-17), 56.8 (CH, C-14), 71.8 (CH, C-3), 121.7 (CH, C-6), 129.2 (CH, C-3), 138.3 (CH, C-22), 140.7 (C, C-5)
β-sitosterol	0.68 (3H, s, H-18), 0.83(3H, d, H-27), 0.84 (3H, d, H-26), 0.90 (3H, s, H-29), 0.93 (3H, d, H-21), 1.01 (3H, s, H-19), 3.54 (1H, m, H-3), 5.35 (1H, dd, H-6)	1.8 (CH3, C-18), 12.0 (CH3, C-29), 18.8 (CH3, C-21), 19.0 (CH3, C-27), 19.4 (CH3, C-19), 19.8 (CH3, C-26), 21.1 (CH2, C-11), 23.1 (CH2, C-28), 24.3 (CH2, C-15), 26.0 (CH2, C-23), 28.2 (CH2, C-16), 29.1 (CH, C-25), 31.6 (CH2, C-2), 31.8 (CH, C-8), 31.9 (CH2, C-7), 33.9 (CH2, C-22), 36.1 (CH, C-20), 36.5 (C, C-10), 37.2 (CH2, C-1), 39.7 (CH2, C-12), 42.3 (CH2, C-4), 42.3 (C, C-13), 45.8 (CH, C-24), 50.1 (CH, C-9), 56.0 (CH, C-17), 56.7 (CH, C-14), 71.8 (CH, C-3), 121.7 (CH, C-6), 140.7 (C, C-5)
***Triterpenoid***		
Lupeol	0.69 (1H, d, H-5), 0.76 (3H, s, H-23), 0.78 (3H, s, H-28), 0.83 (3H, s, H-25), 0.94 (3H, s, H-27), 0.97 (3H, s, H-26), 1.03 (3H, s, H-24), 1.27 (2H, m, H-21), 1.30 (1H, m, H-9), 1.38 (2H, m, H-7), 1.39 (2H, m, H-6), 1.38 (1H, m, H-18), 1.68 (1H, s, H-30), 2.38 (1H, m, H-19), 3.18 (1H, m, H-3), 4.56 (1H, s, H-29b), 4.68 (1H, s, H-29a)	14.5 (CH3, C-27), 15.4(CH3, C-24), 16.0 (CH3, C-26), 16.1 (CH3, C-25), 18.0 (CH3, C-28), 18.3 (CH2, C-6), 19.3 (CH3, C-30), 20.9 (CH2, C-11), 25.1 (CH2, C-12), 27.2 (CH2, C-15), 27.4 (CH2, C-2), 28.0 (CH3, C-23), 29.8 (CH2, C-21), 34.3 (CH2, C-7), 35.9 (CH2, C-16), 37.2 (C, C-10), 38.0(CH, C-13), 38.7 (CH2, C-1), 38.9 (C, C-4), 40.0 (CH2, C-22), 40.8 (C, C-8), 42.8 (C, C-14), 43.0 (C, C-17), 48.0 (CH, C-19), 48.3 (CH, C-18), 50.4 (CH, C-9), 55.3 (CH, C-5), 79.0 (CH, C-3), 109.3 (CH2, C-29), 151.0 (C, C-20)
***Flavonoids***		
Kaempferol	6.20 (1H, d, H-6), 6.44 (1H, d, H-8), 6.93 (1H, d, H-5′), 8.03 (1H, m, H-6′), 9.39 (1H, s, OH), 10.10 (1H, s, OH), 10.78 (1H, s, OH), 12.47 (1H, s, OH)	146.9 (C-2), 135.7 (C-3), 175.9 (C-4), 156.3 (C-5), 98.3(C-6), 163.9 (C-7), 93.6 (C-8), 160.8(C-9), 103.1 (C-10), 121.7 (C-1′),115.5 (C-2′), 129.6 (C-3′), 159.3 (C-4′), 115.5 (C-5′), 121.7 (C-6′)
Isorhamnetin	3.84 (3H, s, OCH3), 6.20 (1H, d, H-6), 6.48 (1H, d, H-8), 6.93 (1H, d, H-5′), 7.70 (1H, m, H-6′), 7.75 (1H, d, H-2′), 9.44 (1H, s, OH), 9.75 (1H, s, OH), 10.76 (1H, s, OH), 12.47 (1H, s, OH)	148.8 (C-2), 135.8 (C-3), 175.9 (C-4), 156.2 (C-5), 98.2(C-6), 163.9 (C-7), 93.6 (C-8), 160.7(C-9), 103.0 (C-10), 121.9 (C-1′),111.7 (C-2′), 146.6 (C-3′), 147.4 (C-4′),115.5 (C-5′), 121.7 (C-6′), 55.8 (OCH3)
Quercetin	6.24 (1H, d, H-6), 6.48 (1H, d, H-8), 6.96 (1H, d, H-5′), 7.63 (1H, m, H-6′), 7.70 (1H, d, H-2′), 12.00 (1H, s, OH)	
